# Small-Bowel Metastatic Melanoma From Primary Mucosal Melanoma of the Anus: A Comprehensive Case Report

**DOI:** 10.14309/crj.0000000000001577

**Published:** 2024-12-27

**Authors:** Andrea Gomez Pons, Frank J. Lukens, Osayande Osagiede

**Affiliations:** 1 Division of Gastroenterology and Hepatology, Mayo Clinic, Jacksonville, FL

**Keywords:** metastatic melanoma, mucosal melanoma, double balloon enteroscopy (DBE), intussusception, small-bowel melanoma, anemia, small-bowel surgery, immunotherapy

## Abstract

Anorectal mucosal melanoma (ARMM) is exceptionally rare, highly malignant, and characterized by a poor prognosis. We present the case of a 76-year-old woman with ARMM and recurrent gastrointestinal (GI) bleeding/anemia caused by small-bowel metastases, which was successfully managed with laparoscopic resection. ARMM is an aggressive type of cancer that has the potential to metastasize to the GI tract approximately 4.5 years after the primary diagnosis. Intussusception and GI bleed are potential complications. Small-bowel metastatic melanoma typically goes undiagnosed until autopsy and requires a multidisciplinary approach. Key treatment options include surgery and immunotherapy to improve patient outcomes.

## INTRODUCTION

Malignant melanoma is a neoplasm with a strong tendency to metastasize to the gastrointestinal (GI) tract, especially the small intestine. Mucosal melanoma (MM), which accounts for <10% of all melanomas, can affect the uvea and mucosal tissues, including the head and neck (55% of cases), anorectum (24%), and vagina (18%). MM is not influenced by ultraviolet radiation, but possibly related to local oxidative stress and/or to immunosuppression.^1^

MM of the anorectal region is an exceptionally rare and highly malignant tumor, accounting for only 0.4%–1.6% of all melanomas and 23.6% of mucosal melanomas.^2,3^ This melanoma subtype is characterized for having an aggressive behavior and poor prognosis. The incidence of anorectal mucosal melanoma (ARMM) increases with age with a mean of 55 years. It is commoner in women and predominantly affects Whites more than African Americans.^2,4^ The overall 5-year survival rate is between 10% and 20%.^2^

Patients are often asymptomatic. When symptoms occur, they may include an anal mass, bleeding, and changes in bowel habits.^2,4^ It is often misdiagnosed and can be mistaken for hemorrhoids, rectal cancer, or adenomatous polyps.^5^ Moreover, lesions can be amelanotic in 20% of cases.^2,4^ The potential for metastasis is high, with the most frequent sites being the inguinal lymph nodes, mesorectum, lungs, liver, bones, and brain.^2^ Sixty-five percent of ARMM are located in the anal canal or transition zone, while the remaining 35% are found in the distal rectum.^4^ Treatment typically includes wide local excision or, in more severe cases, radical abdominoperineal resection; however, the prognosis remains poor despite these interventions.^2^

We present an interesting case of a patient with obscure GI bleeding from small-bowel metastatic melanoma tumors. The aim of our case report was to describe the diagnosis and treatment of small-bowel metastatic melanoma tumors, which is a rare condition.

## CASE REPORT

A 76-year-old woman with a history of *BRAF* wild-type ARMM presented with recurrent GI bleeding and severe iron deficiency anemia of unclear etiology. The patient was diagnosed with ARMM 8 years earlier following a routine colonoscopy which identified a pigmented mass at the anal verge, which was confirmed by biopsy. She underwent surgical excision of the mass, with pathology revealing MM with a depth of 6.0 mm into submucosal tissue, with a tumor size of 0.7 × 0.7 × 0.8 cm. Pathology also showed negative margins, but positive lymphovascular invasion. The patient received adjuvant cisplatin and Temodar. A positron emission tomography/computed tomography (PET/CT) scan at the time of diagnosis showed uptake only at the anal mass.

**Figure 1.**
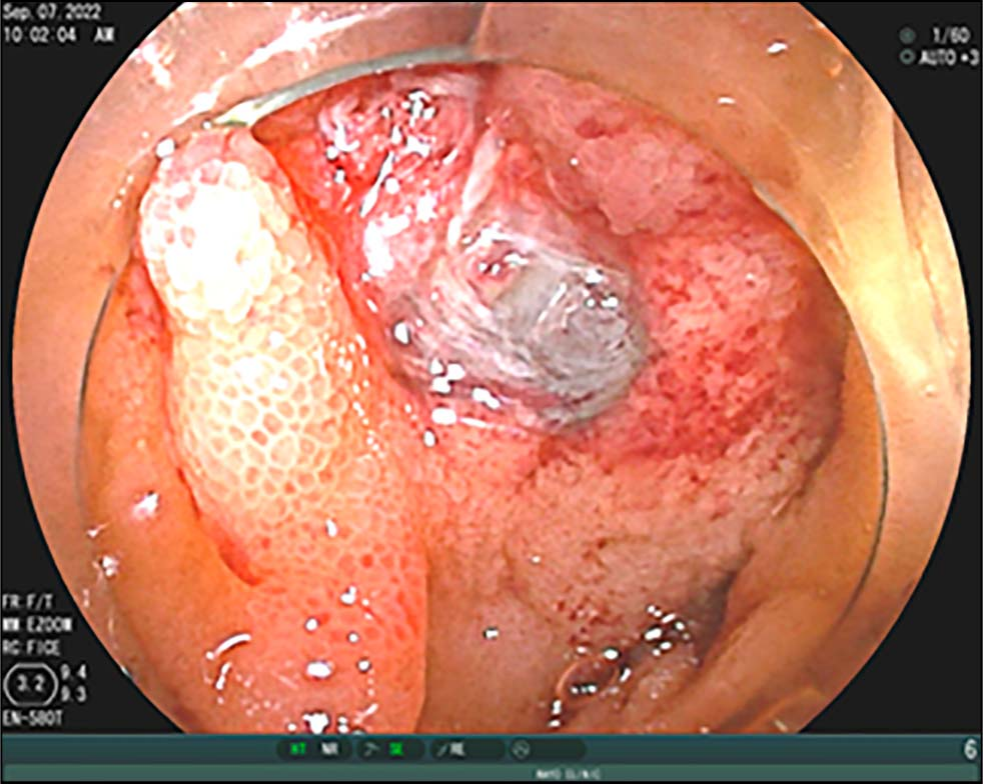
Endoscopic image depicting a dark pigmented submucosal mass in proximal jejunum with surface ulcer.

The patient developed recurrent GI bleeding (melena/dark stools), abdominal pain, and severe anemia 6 years after the initial diagnosis. Subsequent upper and lower double balloon enteroscopy (DBE) identified a dark pigmented submucosal mass in the proximal jejunum, with surface ulceration and nearby blood located 30 cm past the ligament of Treitz (Figure 1). This lesion was biopsied and tattooed at that time. Although pathology results were negative for malignancy, likely due to the submucosal nature of the lesions, the macroscopic characteristics and ulceration raised a strong suspicion of malignancy, and it was suspected that the patient was bleeding from this area. Core needle biopsy of a new hypermetabolic periaortic lymph node noted on PET-CT scan (measuring 3.2 × 2.5 cm) showed metastatic melanoma, *BRAF *wild type. This informed the decision to treat with chemotherapy, immunotherapy (nivolumab/ipilimumab), and Gamma Knife radiosurgery by the oncologist. Small-bowel surgery was considered, but the patient was considered a poor surgical candidate at that time due to worsening of performance status and consideration of hospice care. Fortunately, the patient had significant improvement with systemic therapy. The hospice option was no longer pursued, and the decision was made to continue surveillance with PET-CT scan and MRI.

**Figure 2.**
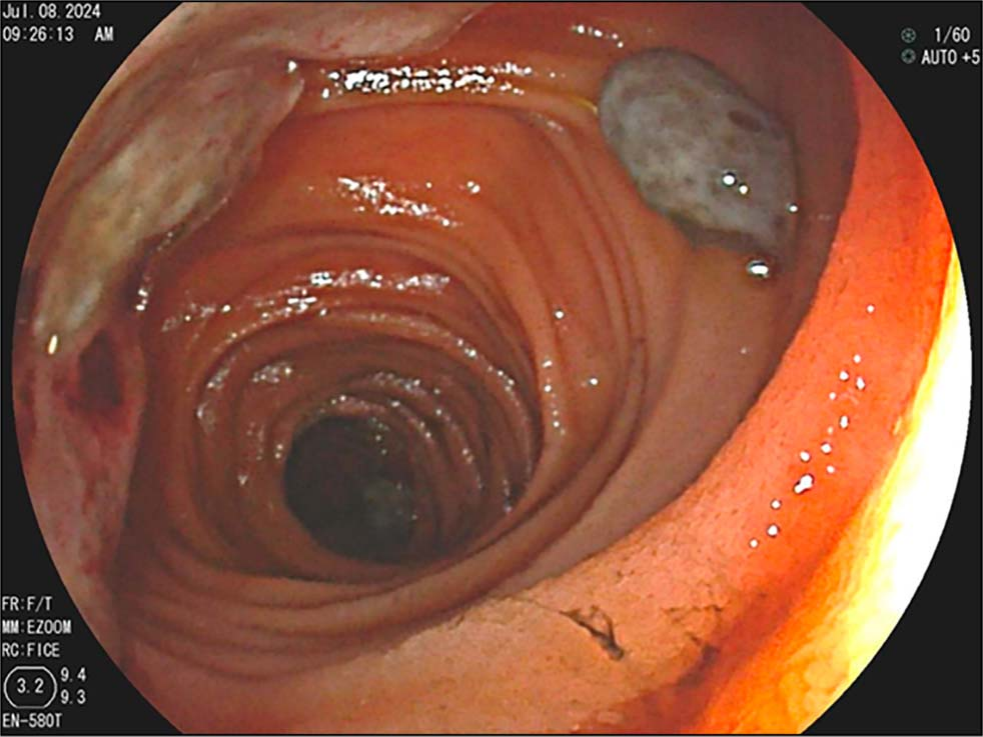
Endoscopic image depicting a brown-colored melanoma tumor from metastatic melanoma in the proximal jejunum.

**Figure 3.**
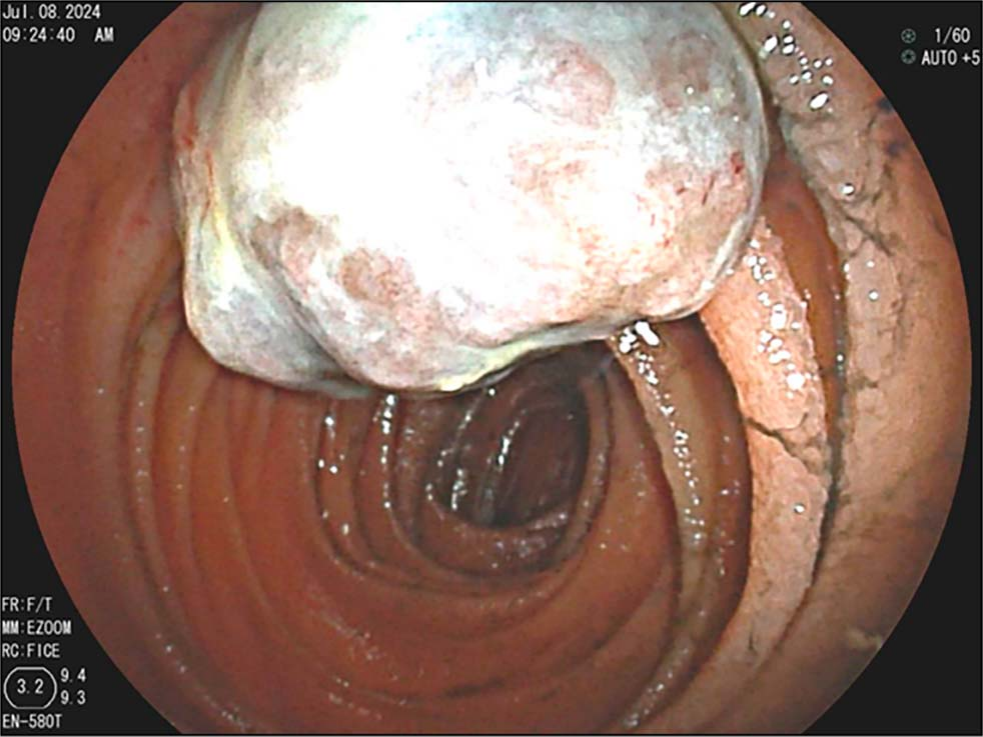
Endoscopic image showing a tumor in the proximal jejunum with a visible tattoo marking. The tumor occupies approximately one-third of the lumen.

**Figure 4.**
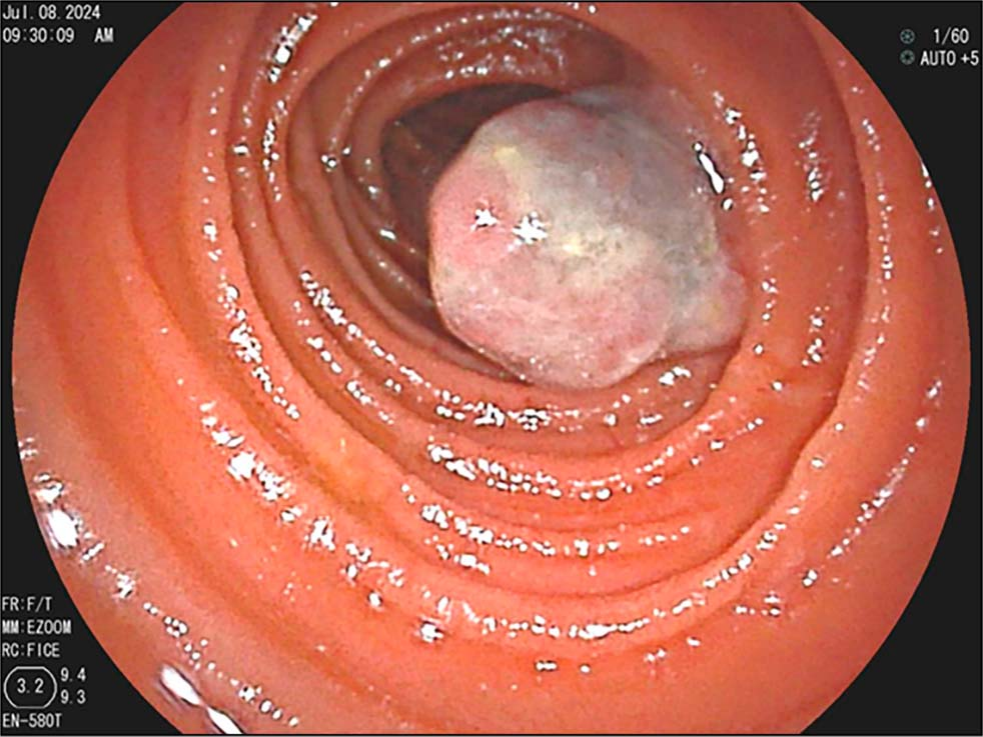
Melanoma tumor in the jejunum, seen with double balloon enteroscopy.

**Figure 5.**
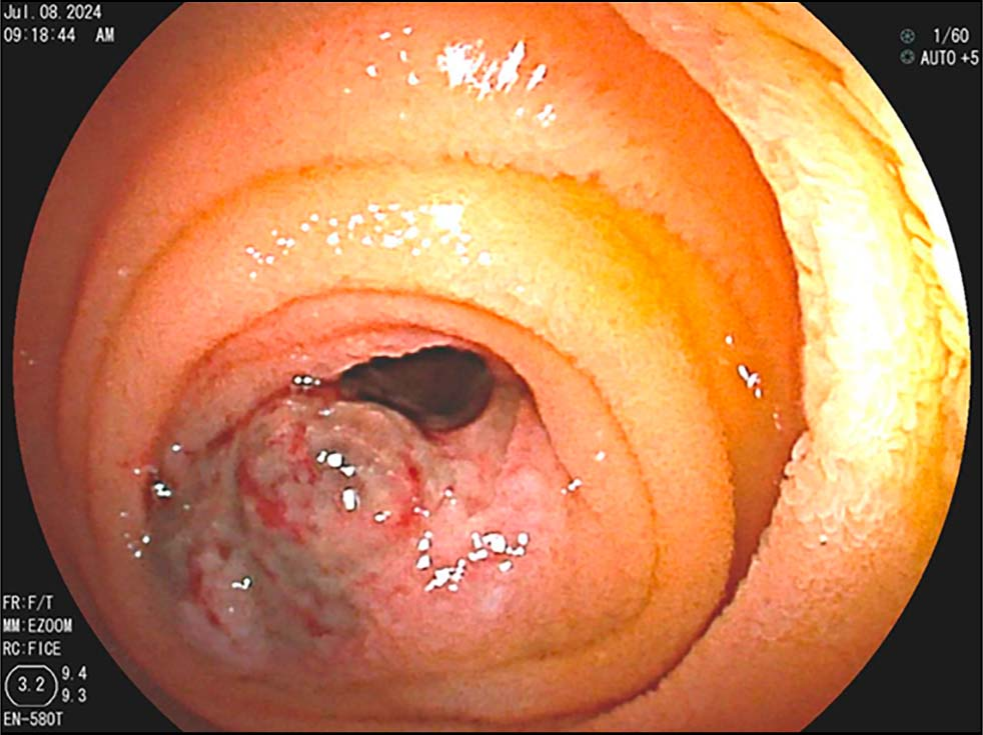
Tumor in the jejunum, approximately 3 cm in size, with ulceration and active blood oozing.

**Figure 6.**
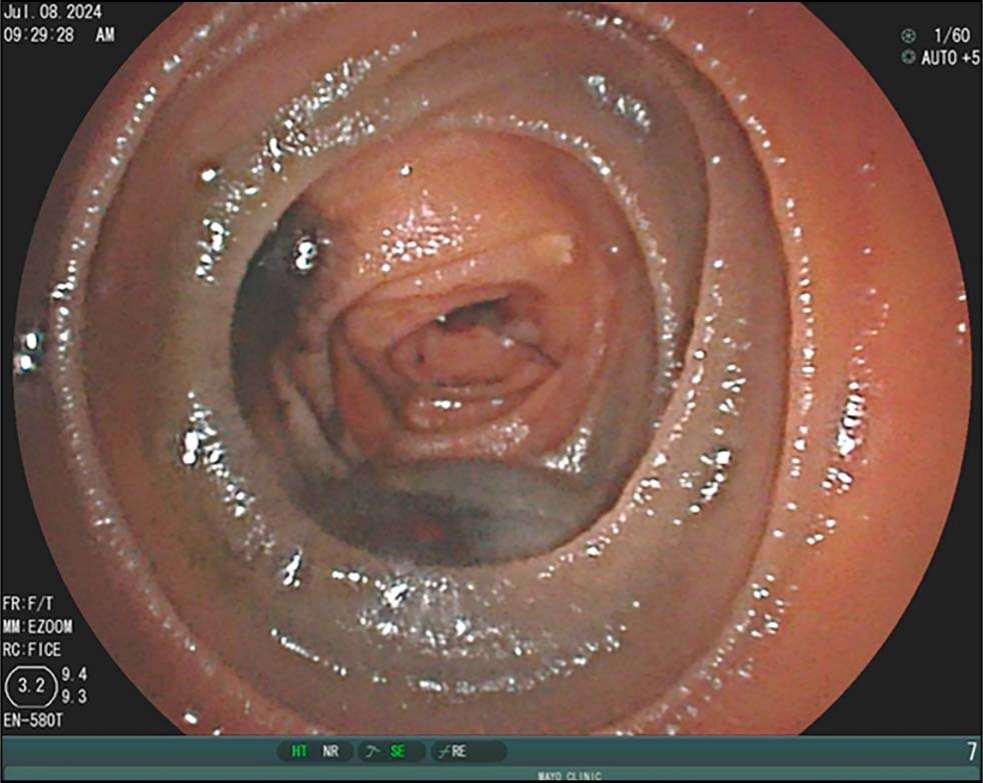
Tattoo injection of the small-bowel proximal and distal-to-metastatic mucosal melanoma tumors.

**Figure 7.**
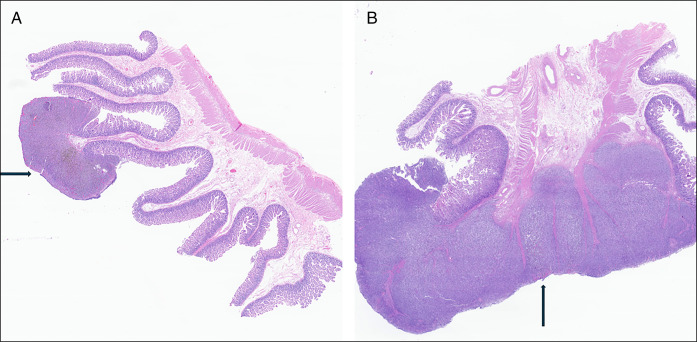
Pathology of the largest mass (right) to include adjacent mesenteric fat (B). A smaller mass is also shown on the left (A).

The patient underwent surveillance PET-CT scan every 2–3 months until a surveillance PET-CT scan 2 years later suggested possible metastatic lesions in the small bowel. The patient was subsequently admitted to the hospital with melenic stools for 3 days, and her hemoglobin level had decreased from 8.5 to 5.3 g/dL. Upper DBE identified 4 submucosal lesions in the proximal jejunum consistent with metastatic disease, the largest measuring 3 cm in diameter with ulceration and signs of bleeding (Figures 2–6). The patient underwent laparoscopic small-bowel tumor resection 2 weeks later, with side-to-side antiperistaltic and stick anastomosis. Remaining bowel measured approximately 400 cm. During the surgery, 6 tumors were identified along with tattoos from previous procedures. Four tumors were clustered in the proximal jejunum, the fifth was located in the distal jejunum and appeared to be causing intermittent intussusception, and the sixth was in the proximal ileum. Surgical pathology showed metastatic melanoma (predominantly epithelioid morphology), forming 4 masses in the proximal jejunum and 2 polypoidal masses in the distal jejunum (involving the mucosa and submucosa and focally extending through muscularis propria), ranging in size from 0.8 cm to 3.3 cm (Figure 7). The margins were negative, with 5 additional lymph nodes negative for metastatic melanoma. Postoperatively, the patient progressed well with return of bowel function on postoperative day 5 and was discharged on postoperative day 6. Surveillance PET-CT scan 1 month after surgery was negative for small-bowel metastases, but showed retrocaval lymph node metastasis, now status postradiation therapy. The patient continues to follow with oncology clinic with continued imaging surveillance every 2–3 months.

## DISCUSSION

ARMM is a highly aggressive malignancy that is difficult to study due to its rarity. Management is challenging because of the tumor's invasive behavior and high recurrence rate.^2^ While small-bowel melanoma typically metastasizes from a primary melanoma tumor, studies have shown that it can also originate de novo in the GI tract.^6^ The estimated time between the diagnosis of the primary lesion and metastasis to the GI tract is approximately 4.5 years.^1^

GI metastasis should be considered in any patient with a history of melanoma presenting with bowel symptoms, GI bleed, or anemia.^7^ Initiation of symptoms often indicates the presence of complications such as perforation, bleeding, obstruction, or intussusception.^8^ Small-bowel bleeding is often the first clinical presentation of small-bowel metastatic melanoma when symptomatic.^6,9^ This often manifests as melena or hematochezia. Chronic intestinal blood losses could also manifest as iron deficiency anemia with a normal upper endoscopy and colonoscopy. DBE is a successful diagnostic strategy; however, capsule endoscopy combined with PET-CT scan could be the first step.^10^

Intussusception is another potential complication of small-bowel melanoma. While it is more commonly seen in the pediatric population, it occurs in only 5% of adults.^11^ In this population, intussusception is typically associated with a pathological lead point, such as a tumor or polyp. Few studies have documented this incidence in small-bowel melanoma.^2,11^ In our patient, intussusception developed primarily due to the size of the lesion. Reduction and resection is a successful procedure with positive outcomes.^2^

Regarding treatment, although MM shares some similarities in mutations and targeted treatments with cutaneous melanoma, it is a molecularly distinct entity.^12^ *BRAF *wild-type ARMM lacks the *BRAF*^V600^ mutation. The treatment of patients with *BRAF *wild-type ARMM is a challenging problem as they do not benefit from treatment with *BRAF *inhibitors.^13^ The most recent approaches focus on immunotherapy, but further investigation is needed to enhance the management.^2,13^ Moreover, free-margin surgery resection has been shown to provide effective palliation and may extend the survival.

In conclusion, 90% of cases of small-bowel metastatic melanoma go undiagnosed until autopsy due to the disease's asymptomatic nature.^8^ DBE is a valuable diagnostic method for identification and biopsying tumors, with minimal complications. Early detection and a multidisciplinary approach are crucial in managing such cases, as timely intervention can improve patient outcomes. Stepwise management is essential, and surgery is recommended, when possible, to help reduce the risk of recurrence. Several studies have demonstrated that immunotherapy is the most effective treatment, with one study describing that combining ipilimumab with a programmed cell death protein 1 inhibitor (nivolumab) yields the highest response rate.^14^ Per current surveillance protocols, PET/CT of the chest/abdomen/pelvis and magnetic resonance imaging/CT brain is recommended every 2–3 months for locoregional or metastatic disease. The surveillance interval can be extended to every 6 months after 2–3 years without disease progression for up to 5 years and then annually for up to 10 years.^15^

Our case highlights the complexities in managing MM and the complications that can arise. Given the severity of our patient's anemia, surgery was deemed the most appropriate treatment, as medical management alone proved insufficient.

## DISCLOSURES

Author contributions: O. Osagiede, FJ Lukens, AG Pons: Conception and design, collection, and interpretation of the clinical data, and drafting of the article. FJ Lukens is the article guarantor.

Financial disclosure: None to report.

Informed consent was obtained for this case report.
